# Socioeconomic status, antidepressant use, and return to work after disability due to common mental disorders

**DOI:** 10.1192/j.eurpsy.2025.10046

**Published:** 2025-06-13

**Authors:** Helena Leppänen, Olli Kampman, Reija Autio, Antti Tanskanen, Heidi Taipale, Tino Karolaakso, Päivi Rissanen, Turkka Näppilä, Sami Pirkola

**Affiliations:** 1 Faculty of Medicine and Health Technology, Tampere University, Tampere, Finland; 2 Department of Psychiatry, The Wellbeing Services County of Pirkanmaa, Finland; 3 Department of Clinical Sciences, Psychiatry, Umeå University, Umeå, Sweden; 4 Faculty of Medicine, Department of Clinical Medicine (Psychiatry), University of Turku, Finland; 5 Department of Psychiatry, The Wellbeing Services County of Ostrobothnia, Finland; 6 Faculty of Social Sciences, Tampere University, Tampere, Finland; 7 Niuvanniemi Hospital, Kuopio, Finland; 8 Department of Clinical Neuroscience, Karolinska Institutet, Center for Psychiatry Research, Stockholm, Sweden; 9 School of Pharmacy, University of Eastern Finland, Kuopio, Finland; 10 Tampere University Library, Tampere University, Tampere, Finland

**Keywords:** antidepressants, common mental disorders, disability pension, return to work, socioeconomic status

## Abstract

**Background:**

Common mental disorders (CMDs) are significant causes of work disability. Low socioeconomic status (SES) is a known risk factor for CMDs and work disability, one possible reason being poorer treatment adherence. We aimed to study the realization of pharmacological treatment and antidepressant adherence in patients with CMDs 3 years before and 3 years after being granted a disability pension (DP) and the role of SES in this. We also studied whether antidepressant adherence is associated with return to work (RTW) after a temporary DP.

**Methods:**

Information on all persons granted a DP due to CMD between 2010 and 2012 in Finland (*n* = 12,388) was retrieved from national registers, which included medical, socioeconomic, and sociodemographic information of the subjects. We used the PRE2DUP method to estimate drug use periods and regression analyses to study associations between SES, taking medications, and RTW.

**Results:**

Prevalence of taking antidepressants increased towards the DP grant and decreased thereafter, but 14.6% of subjects did not take antidepressants or antipsychotics at all during the study period. Of SES factors, only income was positively associated with antidepressant adherence, lasting over a year. Antidepressant adherence was not associated with RTW.

**Conclusions:**

An alarming result was the absence of recommended medication in fewer than every seventh patient estimated to be disabled due to pharmacologically treatable psychiatric disorders. Contrary to expectations, SES had only a minor predictive role in antidepressant adherence in this patient group. Contrary to taking antidepressants, rehabilitation was associated with RTW. The results adduced the importance of CMD treatment optimization regardless of SES.

## Introduction

Common mental disorders (CMDs) are extremely prevalent and common causes of work disability [1–3]. CMDs include depression, generalized anxiety disorder, social anxiety disorder, panic disorder, phobias, obsessive-compulsive disorder, and post-traumatic stress disorder (PTSD) [4]. Psychotherapy and pharmacotherapy are evidence-based treatments for CMDs [4, 5]. The relationship between CMDs and work disability is complex, often requiring diverse measures to support working ability [1, 6]. Although the burden of work disability outcomes due to CMDs for individuals and societies is globally recognized, they remain widely undertreated [7].

Low socioeconomic status (SES) has been shown to be linked to the development of CMDs and psychiatric work disability or less frequent return to work (RTW) [8–11]. Patient-related factors, such as higher self-efficacy and conscientiousness, positively predict RTW, while older age, male gender, and neuroticism have the opposite effect [10]. Psychotherapy and other rehabilitation may shorten work disability and be conducive to RTW [8, 12–14].

Antidepressants are efficacious in treating CMD symptoms [4, 6, 15], and in treatment-resistant depression, combinations of antidepressants or antidepressants and antipsychotics are preferable to monotherapy [16, 17]. After an initial response, antidepressants are recommended to be continued for several months to prevent relapse [18]. According to systematic reviews, continuous antidepressant treatment lasting up to a year prevents relapses in both depressive [19] and anxiety disorders [20]. Whether antidepressants are indeed conducive to RTW is unclear [13, 21, 22]. The use of antidepressants has even been shown to relate to a poorer prognosis, probably due to more active prescribing for people with more severe symptoms [23]. Benzodiazepine medication, on the other hand, may impair antidepressant efficacy and the patient’s function in depression [24]. Taking benzodiazepines for over 4 weeks has not been proven efficient in treating depression [25] and has been shown to be associated with labour market marginalization [26].

Evidence suggests that SES modifies the use of pharmacological treatment of CMDs. Australian [27], British [28], and Nordic [29, 30] studies have shown that people with lower SES are more likely to take antidepressants. By contrast, a Finnish study reported contrasting results, but with a study population consisting of government employees, which was thus not representative of the population as a whole [31]. People with lower SES may use fewer recently marketed medications [32, 33], but opposite results have also been reported [30]. On the other hand, more benzodiazepines and related drugs (BZDR), carrying a risk of dependency in long-term use, are prescribed to people with lower SES, who may be more prone to taking benzodiazepines long term [34–36].

Adherence is defined as the degree to which a patient’s behaviour complies with recommended health advice [37]. Non-adherence to psychiatric medication is high and estimates vary from 10 to 60% in mood disorders [38]. Of SES factors, research has found a lower level of education [37–40], unemployment [38, 40], and lack of money [38] to be risk factors for non-adherence. Of sociodemographic factors, male gender, non-marital family status, and younger age have been shown to associate with non-adherence, with mixed evidence [37, 38].

To sum up, CMDs are still frequently undertreated despite the heavy burden they impose on work disability outcomes. Low SES is a known risk factor in the development of CMDs, psychiatric work disability, and less frequent RTW. Antidepressants may be more commonly used among low SES patients, but adherence may be lower among them. With this study of CMD patients granted a disability pension (DP), we aim to explore medication use before and after the granting of the DP and to elucidate the role of SES in medication adherence and its association with RTW. Based on earlier research, we hypothesize that (1) taking antidepressants remains suboptimal at the group level, (2) lower SES is associated with poorer antidepressant adherence and long-term BZDR use, (3) antidepressant adherence is positively or not at all associated with RTW, and (4) long-term BZDR use is negatively associated with RTW.

## Data and methods

### Study population

The study is a part of the RETIRE research project, which identifies risk factors for psychiatric DPs and measures to prevent premature retirement [8, 14, 41]. The dataset included all Finnish citizens granted a temporary or permanent DP for the first time between 2010 and 2012 due to CMD diagnoses (ICD-10: F32–F34 and F40–43, *N* = 12,388). Out of 12,388 retirees, 4,492 (35.9%) were granted a temporary DP, whereas the others were granted a permanent DP. Of the temporary DP recipients, 1,315 (29.3%) returned to work during the 3-year follow-up beginning from the granting of the DP, whereas 646 (14.4%) returned to work partially, and 2,531 (56.3%) not at all.

Besides the main CMD diagnosis, possible second and third diagnoses were also recorded and counted as comorbidities. The following comorbidities (with >40 cases) were recorded: dementia, organic brain syndrome, or intellectual disability (ICD-10: F01–03, F04–09 and F70–79); substance abuse (F10–19); psychotic disorders (F20–29); personality disorders (F60–61); autism (F84); attention-deficit hyperactivity disorder (F90); musculoskeletal disorders (M00–99); and other somatic conditions, except musculoskeletal disorders (A00–B99, C00–D48, D50–89, E00–90, G00–99, H00–95, I00–99, J00–99, K00–93, L00–99, N00–99, and S00-T98). Frequencies of comorbidities are shown in Supplementary Table I.

We obtained data from the registers of the Finnish Centre for Pensions, the National Institute for Health and Welfare, the Social Insurance Institution, and Statistics Finland. These registers supplied the study subjects’ medical, socioeconomic, and sociodemographic information.

### Socioeconomic and sociodemographic factors

The socioeconomic and sociodemographic factors have been described in more detail elsewhere [42, 43] and are summarized here. Factors representing dimensions of SES were income, education, and occupational status. Average annual disposable income of a household was calculated by consumption unit (defined by Organisation for Economic Co-operation and Development) for each study subject and divided into quintiles: lowest (<14,454 euros), middle-lower (14,455e–20,468e), middle (20,469e–25,931e), middle-higher (25,932e–33,254e), and highest (more than 33,255e) [44]. Education was categorized into five levels: basic level, upper secondary, short-cycle tertiary, lower degree tertiary, and higher degree tertiary education. Occupational status was classified into six groups: student, unemployed, blue-collar worker, lower and upper white-collar workers, and entrepreneur. Sociodemographic factors were gender (female/male), age categorized into five groups (18–25, 26–35, 36–45, 46–55, and 56–65 years), and family type categorized into four groups (living alone, couple, single parent, and couple with children). Sociodemographic and socioeconomic factors were measured on the last day of the year before the commencement of the DP.

### Medication use

The medication records of each study subject were monitored from 3 years before to 3 years after the date on which DP was granted. Medications dispensed were derived from the prescription register. Medications of interest were antidepressants with ATC code N06A. Antidepressants were sub-categorized into Selective Serotonin Reuptake Inhibitors (SSRIs, with ATC code N06AB), Serotonin-Norepinephrine Reuptake Inhibitors (SNRIs, with ATC codes N06AX16, N06AX17, and N06AX21), mirtazapine (N06AX11), and other antidepressants also including lithium (N05AN01). Medications studied were also antipsychotics (N05A except lithium), and BZDR divided to anxiolytics (N05B, N3AE01, and N06CA01) and hypnotics (N05CD and N05CF) [45]. Medications are listed in detail in Supplementary Table II, and medications taken during the 6-year study interval are described in detail in Supplementary Tables III and VI.

Considering the study population with prolonged or recurrent mental health conditions, we determined that antidepressants should be taken for at least a year to prevent relapse. Antidepressant adherence in this article refers to antidepressants taken continuously for over a year. On the other hand, benzodiazepines are not recommended for chronic use, and in our study, their use is considered long-term when exceeding 6 months.

### Compensation for medications

Reimbursement of the costs of medications through the Finnish social insurance system is generally 40% for antidepressants. For those suffering from psychotic depression, the compensation is 100% with a personal responsibility of 4.5 euros per purchase. The maximum annual personal responsibility is around 630 euros [6, 46].

### Rehabilitation

In Finland, rehabilitative psychotherapy is provided for 1–3 years, and vocational rehabilitation may also last for several years, for example, in the form of vocational training [47]. Rehabilitative psychotherapy and vocational rehabilitation were measured as binary variables, from 5 years before and 3 years after the DP year.

### Work disability and RTW

In the Finnish disability retirement system, when the working ability of a person between 18 and 64 years is reduced due to physical illness, mental health condition, handicap, or injury, sickness allowance is granted for a maximum of 1 year [48]. If the disability persists but the person is potentially able to return to work through rehabilitation, a temporary DP may be granted for a fixed period, which must be re-assessed at the end of the renewal period. If no potential for regaining one’s working ability is seen, a permanent DP is granted. Assessment of the DP grant is based on a medical statement and the person’s ability to study or obtain income through work, considering the applicant’s age, place of residence, previous education, working history, and job requirements. The DP is paid on the basis of an earnings-related scheme or the national pension scheme, which guarantees a minimum income level [48]. The RETIRE research project has identified three clusters of patterns of returning to work from the temporary DP during the 3-year follow-up: full RTW, partial RTW, and no RTW [8]. In the full RTW group, individuals returned to work during the first 2 years after temporary DP and continued at work during the 3-year follow-up. In the partial RTW group, individuals only returned to work for a short period, usually 1 year during the follow-up time. In the no RTW group, individuals continued DP.

### Statistical methods

Drug use periods were estimated using the PRE2DUP method, by which sliding averages of defined daily doses were calculated [49]. This method takes account of periods of hospitalization, stockpiling of medication, changing doses, and personal purchasing patterns. Antidepressant and BZDR use periods were estimated for each person from 3 years before to 3 years after the DP granting date.

Binary and multinomial logistic regression analyses were used to study the likelihood of long-term antidepressant and BZDR use. Outcome variables in the analyses were antidepressant use both as a binary variable and categorized as none, 1–90 days, 91–365 days, and over 365 days sustained use during the study period, and BZDR (including anxiolytics and hypnotics) use categorized as none, 1–180 days, and over 180 consecutive days. Explanatory variables were sociodemographic factors (gender, age, and family status), socioeconomic factors (education, income, and occupational position), rehabilitation (psychotherapy and occupational rehabilitation), comorbidities, and type of DP (temporal or permanent).

Multinomial logistic regression analyses were also used to study the association between long-term medication use and RTW. The outcome variable RTW was categorized as no RTW, partial RTW, and full RTW, and the explanatory variables were the same as in the previous models but additionally included the duration of antidepressant and BZDR use. Both crude and adjusted odds ratios (ORs) with 95% confidence interval (95% CI) were computed with regression analyses. *χ*^2^ tests were used to analyse the associations between the categorical variables.

Prevalences of different medication groups were defined during a specific day on a semi-annual basis, based on existing medication use periods. Taking medications in different medication groups was also modelled using Poisson interrupted time series analysis (ITSA) over a period of 3 years before and after retirement. The number of recipients for each medication group was the outcome variable, which was modelled using time and retirement as exposure variables, with retirement acting as a changepoint in the model.

In all our analyses, *p* < 0.05 was considered statistically significant. The statistical analyses were carried out using IBM SPSS Statistics for Windows, version 28 (IBM, Armonk, NY) and R 4.3.2 (The R Foundation), with package ggplot2 [50].

## Results

### Descriptive analysis

**Figure 1.**
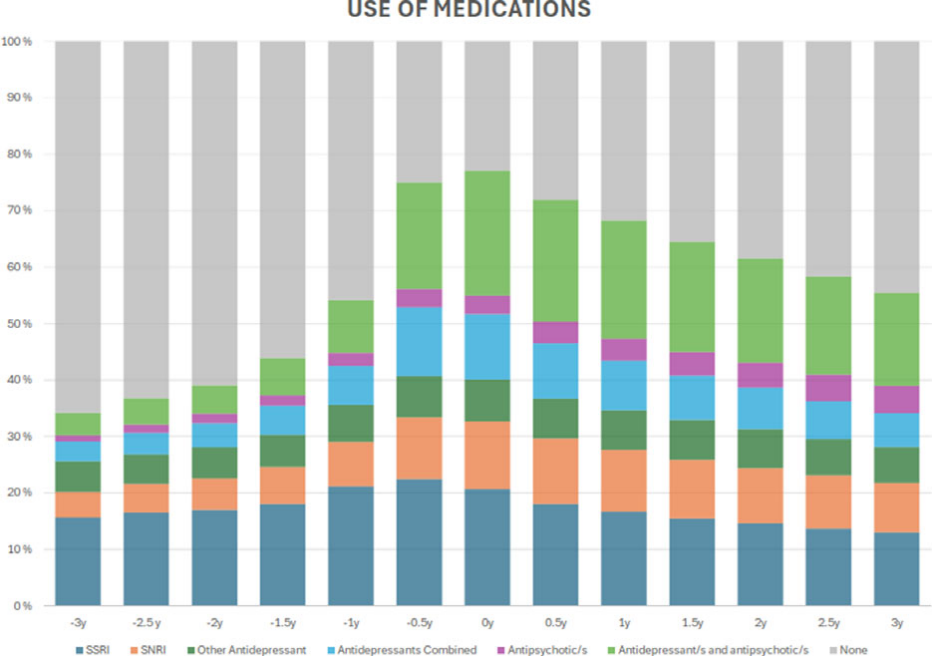
Prevalence of the medications taken from 3 years before to 3 years after the DP among patients with CMD.

The mean age of the study population was 47 years (range: 18–65 years), and 62.2% were women. Antidepressants were used by 34.2% 3 years before DP, by 54.1% 1 year before DP, and by 77.1% in the DP year. After DP, antidepressant use decreased gradually, and antidepressants were used by 55.5% of the patients 3 years after DP (Figure 1). The most frequently taken antidepressant group was SSRIs, and the second was SNRIs. The combination of antidepressant/s and/or antipsychotic/s increased from 7.5% 3 years before DP to 33.7% in the DP year and then decreased to 22.5% by the end of the study period (Figure 1). The use of each medication group before and after DP is shown in Supplementary Figure I.

The main CMD diagnoses were, in most cases, depressive disorders (89.9%), and the remainder were anxiety disorders (10.1%) (Table 1). No additional comorbid diagnosis is needed to justify a DP. However, 68.3% of our study subjects had a second diagnosis, and 5.6% had a third diagnosis indicating comorbidities in the DP applications. Of the comorbidities, 3,533 (41.5%) were other CMDs, mostly anxiety disorders, whereas 2,340 (27.5%) were other mental health conditions, 1,353 (15.9%) somatic conditions (excluding musculoskeletal disorders), and 1,285 (15.1%) musculoskeletal disorders.

**Table 1.** Main CMD diagnosis and prevalence of antidepressants, antipsychotics, anxiolytics, and hypnotics taken during the 6-year study period

Main CMD diagnosis	Total	%	Antidepressants in use (n/%)	Antipsychotics in use (n/%)	Anxiolytics in use (n/%)	Hypnotics in use (n/%)
Major depressive disorder	5,452	44.0	4,412 (80.9)	3,048 (55.9)	2,402 (44.1)	2,128 (39.0)
Major depression, recurrent	5,385	43.5	4,285 (79.6)	2,976 (55.3)	2,318 (43.0)	2,065 (38.3)
Cyclothymia	295	2.4	248 (84.1)	162 (54.9)	137 (46.4)	118 (40.0)
Phobic anxiety disorder	270	2.2	216 (80.0)	156 (57.8)	116 (43.0)	109 (40.0)
Other anxiety disorder	617	5.0	501 (81.2)	325 (52.7)	251 (40.7)	237 (38.4)
Obsessive–compulsive disorder	173	1.4	138 (79.8)	103 (59.5)	66 (38.2)	69 (39.9)
Reaction to severe stress and adjustment disorders	196	1.6	152 (77.6)	105 (53.6)	80 (40.8)	71 (36.2)
Total	12,388	100	9,952 (80.3)	6,875 (55.5)	5,370 (43.3)	4,797 (38.7)

In all, 2,436 subjects (19.7%) did not take antidepressants at all during the 6-year time interval around the DP. Of these, 626 (25.7%) took antipsychotics, of which the most common was quetiapine (43.3%), 417 (17.1%) received psychotherapy, and 948 (38.9%) received vocational rehabilitation (Supplementary Table I). However, 1,810 subjects (14.6%) were not on any antidepressants or antipsychotics, and 984 subjects (7.9%) were not on any above-mentioned medications or involved in rehabilitation.

Mean antidepressant total use (including all antidepressant use periods of the subjects) during the 6-year study period (2,193 days) was 1,084 days (SD = 735), and 396 persons (3.2%) used antidepressants for the maximum time, that is, 6 consecutive years. The mean longest individual period for taking antidepressants was 904 days (SD = 708). Altogether, 5,370 (43.3%) subjects took anxiolytics, and 4,797 (38.7%) took hypnotics during the study period. Mean anxiolytics total use was 701 days (SD = 744), and mean hypnotics total use was 607 days (SD = 692).

Those taking antidepressants had an average of 2.7 (range: 1–11) different antidepressants during the study period, and 26.8% took one, 27.2% took two, and 46.0% took three or more different antidepressants. In total, 6,875 study subjects (55.5%) took on average 1.9 different antipsychotics (range: 1–10), of which 51.4% took one, 24.4% took two, and 24.2% took three or more different antipsychotics. Furthermore, 7,282 subjects (58.8%) took BZDR with a mean of 1.9 (range: 1–8) different drugs, and 48.8% took one, 27.8% took two, and 23.3% took three or more different BZDR. Given the wide individual variation in medications taken, no associations between SES and different medications or combinations could be further analysed.

### Medications taken before and after DP

Poisson ITSA over a period of 3 years before and after retirement was used to model medications taken (Supplementary Figure I). For all medications, except antipsychotics, the trend in taking the medication shifted from increasing to decreasing after the DP. In contrast, for antipsychotic monotherapy, the rising trend slowed but remained positive even after the DP. For each medication, the model showed a statistically significant change in the slope of the trend after the DP (*p* < 0.0001).

### Association of socioeconomic and sociodemographic factors with medication use and adherence

We investigated more thoroughly those not taking antidepressants at all during the study period. Taking antidepressants, defined as one or more purchases during the 6-year study period, varied between 77.6% in PTSD and 84.1% in cyclothymia (Table 1). No statistically significant differences were found between the CMD diagnoses and taking antidepressants. Neither did the binary logistic regression model explain the relationships between taking antidepressants and comorbidities, SES, sociodemographic factors, or rehabilitation (data not shown).

The longest individual periods of taking antidepressants were categorized as 1–90 days (6.7%), 91–365 days (17.1%) and over a year (56.7%), and no antidepressants at all (19.7%), which was used as the reference category in the logistic regression. Supplementary Table I describes the socioeconomic and sociodemographic factors of the study population in terms of categories of taking antidepressants. In the final multinomial logistic regression model (Table 2), only the highest income category was positively associated with antidepressant adherence lasting over a year (OR 1.30, 95% CI 1.04–1.62). Similar analyses were conducted for taking anxiolytics and hypnotics, but with categories: none, 1–180 days, and over 180 days, and no associations were found (Supplementary Tables VII and VIII).

**Table 2.** Socioeconomic and sociodemographic factors and rehabilitation associated with the maximum duration of taking antidepressants, modelled with multinomial logistic regression models. The reference is none

	1–90 days				91–365 days				Over a year			
	Crude model		Final model		Crude model		Final model		Crude model		Final model	
	OR	95% CI	OR	95% CI	OR	95% CI	OR	95% CI	OR	95% CI	OR	95% CI
Gender												
Male	1.02	0.86–1.20	1.00	0.82–1.21	**1.14**	**1.01–1.29**	**1.17**	**1.01–1.35**	1.06	0.96–1.16	1.05	0.93–1.18
Female (reference)	1		1		1		1		1		1	
Age												
18–25 years	0.90	0.65–1.24	0.95	0.58–1.54	0.81	0.65–1.02	0.88	0.62–1.24	0.90	0.75–1.08	1.04	0.79–1.37
26–35 years	1.06	0.81–1.38	1.14	0.77–1.69	0.85	0.70–1.04	0.96	0.72–1.27	0.97	0.83–1.14	1.18	0.94–1.48
36–45 years	1.03	0.82–1.30	1.12	0.81–1.53	**0.72**	**0.60–0.86**	0.81	0.64–1.03	**0.86**	**0.75–0.98**	0.92	0.77–1.11
46–55 years	1.07	0.87–1.30	1.15	0.90–1.45	0.86	0.75–1.00	0.84	0.71–1.01	0.94	0.84–1.06	0.96	0.84–1.11
56–65 years (reference)	1		1		1		1		1		1	
Family status												
Living alone	1.22	0.98–1.52	1.14	0.88–1.47	1.00	0.85–1.16	1.01	0.84–1.21	1.02	0.90–1.15	1.05	0.90–1.21
Couple	**1.28**	**1.02–1.60**	**1.31**	**1.01–1.69**	1.10	0.93–1.29	1.03	0.85–1.24	1.03	0.90–1.17	0.99	0.85–1.14
Single parent	**1.52**	**1.14–2.02**	**1.45**	**1.05–2.01**	0.88	0.70–1.10	0.95	0.74–1.22	0.96	0.81–1.15	1.01	0.83–1.23
Couple with children (references)	1		1		1		1		1		1	
Education												
Higher degree tertiary	1.19	0.84–1.68	1.06	0.68–1.64	1.06	0.82–1.37	0.92	0.66–1.29	1.04	0.84–1.28	0.94	0.72–1.23
Lower degree tertiary	1.14	0.80–1.62	1.12	0.74–1.68	1.10	0.84–1.42	0.96	0.70–1.31	1.23	1.00–1.51	1.17	0.92–1.49
Short-cycle tertiary	1.19	0.92–1.54	1.14	0.83–1.56	1.21	1.00–1.46	1.12	0.89–1.41	1.10	0.95–1.29	1.07	0.89–1.29
Upper secondary level	1.08	0.90–1.31	1.07	0.84–1.35	1.03	0.90–1.19	0.98	0.83–1.16	1.08	0.97–1.21	1.11	0.97–1.27
Basic level (reference)	1		1		1		1		1			
Income												
Highest	1.25	0.97–1.62	1.18	0.81–1.71	**1.34**	**1.10–1.63**	1.16	0.89–1.53	**1.35**	**1.15–1.58**	**1.30**	**1.04–1.62**
Middle-higher	1.00	0.78–1.29	0.97	0.69–1.36	1.08	0.90–1.30	0.96	0.75–1.23	1.11	0.96–1.29	1.05	0.86–1.28
Middle	0.92	0.73–1.17	0.92	0.67–1.25	**1.22**	**1.03–1.45**	1.09	0.87–1.37	1.09	0.95–1.25	1.01	0.84–1.21
Middle-lower	0.96	0.77–1.20	0.97	0.73–1.30	1.03	0.87–1.21	0.94	0.76–1.17	1.05	0.92–1.19	0.99	0.84–1.18
Lowest (reference)	1		1		1		1		1		1	
Occupation												
Entrepreneur	0.92	0.62–1.36	0.89	0.60–1.33	1.19	0.91–1.55	1.16	0.88–1.52	0.99	0.80–1.23	0.98	0.79–1.23
Upper white-collar worker	1.33	0.98–1.79	1.28	0.90–1.84	1.03	0.82–1.30	1.00	0.76–1.32	1.06	0.89–1.27	1.02	0.82–1.27
Lower white-collar worker	1.11	0.87–1.42	1.07	0.82–1.39	1.00	0.84–1.19	0.99	0.82–1.19	1.02	0.89–1.18	1.00	0.86–1.16
Student	1.05	0.75–1.47	1.13	0.76–1.68	0.86	0.67–1.10	0.90	0.67–1.21	0.94	0.78–1.15	0.99	0.78–1.24
Unemployed	1.05	0.77–1.44	1.05	0.75–1.47	0.94	0.75–1.18	0.96	0.75–1.23	0.87	0.73–1.04	0.88	0.73–1.07
Blue-collar worker (reference)	1		1		1		1		1		1	
Comorbidity												
Somatic condition (excluding musculoskeletal disorders)	1.09	0.85–1.40	1.16	0.88–1.55	1.02	0.84–1.23	0.93	0.75–1.16	1.07	0.92–1.24	1.08	0.91–1.29
Musculoskeletal disorder	1.13	0.88–1.47	0.99	0.73–1.34	1.12	0.93–1.36	0.99	0.79–1.24	1.11	0.95–1.30	1.00	0.84–1.91
Psychotic disorder	1.32	0.62–2.80	1.40	0.53–3.74	1.00	0.54–1.85	1.41	0.67–2.95	1.17	0.73–1.89	1.30	0.70–2.40
Bipolar disorder	2.08	0.66–6.56	1.96	0.32–11.82	0.99	0.33–2.95	1.29	0.39–4.25	1.34	0.58–3.09	1.20	0.45–3.26
ADHD	1.66	0.48–5.68	3.01	0.60–15.05	1.32	0.48–3.65	1.88	0.45–7.93	1.49	0.65–3.40	2.69	0.80–8.99
Autism	1.05	0.33–3.32	0.71	0.15–3.33	0.95	0.39–2.28	0.66	0.22–2.01	0.69	0.34–1.44	0.65	0.29–1.48
Substance abuse	1.17	0.74–1.85	0.87	0.46–1.65	1.00	0.70–1.43	1.03	0.66–1.60	1.05	0.79–1.39	0.83	0.57–1.20
Personality disorder	0.91	0.73–1.15	0.97	0.74–1.29	0.99	0.84–1.17	1.08	0.89–1.32	0.96	0.84–1.09	1.00	0.85–1.17
Dementia, organic brain syndrome, and intellectual disability	0.83	0.33–2.05	0.79	0.26–2.41	**0.44**	**0.19–0.99**	**0.30**	**0.10–0.91**	0.91	0.55–1.51	0.75	0.40–1.40
Vocational rehabilitation	1.08	0.92–1.26	1.09	0.90–1.30	1.03	0.91–1.16	1.06	0.92–1.21	1.03	0.94–1.13	1.03	0.92–1.14
Psychotherapy	0.88	0.71–1.09	0.82	0.64–1.06	0.99	0.85–1.16	1.03	0.86–1.24	0.97	0.86–1.10	0.96	0.83–1.11
Permanent DP	1.00	0.85–1.17	0.99	0.79–1.25	1.00	1.00–1.28	1.00	0.84–1.19	1.11	1.01–1.22	1.12	0.97–1.28

*Note:* The reference category for the outcome is no antidepressant use.

Total *n* = 9,074 in the final multinomial logistic regression model.

Final model is adjusted with all the factors in the table.

Statistical significance *p* < 0.05 (bolded).

### Association of taking medications and comorbidities with RTW

Table 3 shows associations of taking medications, rehabilitation, and comorbidities with RTW after temporary DP due to CMDs. The final regression model has been adjusted for socioeconomic and sociodemographic factors and all the factors in the table. No associations were found between periods of taking antidepressants lasting over a year, or between different periods of taking BZDR, and RTW. On the other hand, rehabilitative psychotherapy and vocational rehabilitation were both positively associated with RTW. Of comorbidities, personality disorders were negatively associated with RTW.

**Table 3.** Associations between maximum duration of taking medications, rehabilitation, and comorbidities with RTW in multinomial logistic regression models

	RTW				Partial RTW			
	Crude model		Final model		Crude model		Final model	
	OR	95% CI	OR	95% CI	OR	95% CI	OR	95% CI
Antidepressant use (reference = no use)								
1–90 days	0.99	0.74–1.32	1.06	0.73–1.54	1.06	0.74–1.52	1.09	0.71–1.68
91–365 days	0.91	0.73–1.13	0.86	0.65–1.13	0.84	0.63–1.11	0.76	0.55–1.06
Over a year	0.98	0.82–1.16	0.98	0.78–1.22	0.90	0.71–1.11	0.85	0.65–1.10
Anxiolytic use (reference = no use)								
1–180 days	0.85	0.71–1.02	0.79	0.61–1.01	0.91	0.72–1.16	0.80	0.59–1.08
Over 180 days	0.92	0.79–1.09	0.85	0.68–1.06	0.99	0.81–1.23	0.89	0.68–1.17
Hypnotic use (reference = no use)								
1–180 days	0.88	0.74–1.05	1.00	0.79–1.27	1.06	0.86–1.32	1.19	0.90–1.58
Over 180 days	0.92	0.77–1.10	0.98	0.77–1.25	0.80	0.63–1.02	0.87	0.64–1.18
Vocational rehabilitation	**1.78**	**1.54–2.04**	**1.41**	**1.19–1.66**	**1.67**	**1.40–2.00**	**1.48**	**1.21–1.81**
Psychotherapy	**1.55**	**1.33–1.80**	**1.23**	**1.01–1.49**	**1.50**	**1.23–1.81**	1.22	0.96–1.53
Comorbidity								
Somatic condition (excluding musculoskeletal disorders)	1.09	0.86–1.40	0.84	0.62–1.15	0.89	0.63–1.24	0.82	0.55–1.23
Musculoskeletal disorder	1.12	0.85–1.49	0.80	0.55–1.15	0.90	0.61–1.34	0.91	0.56–1.47
Psychotic disorder	**0.44**	**0.20–0.94**	0.71	0.29–1.73	1.12	0.55–2.28	1.26	0.54–2.95
Bipolar disorder	0.27	0.06–1.21	0.19	0.02–1.61	1.12	0.37–3.41	1.35	0.39–4.60
ADHD	0.46	0.19–1.12	0.60	0.20–1.81	0.31	0.07–1.32	0.43	0.10–1.93
Autism	**0.18**	**0.06–0.60**	0.29	0.07–1.27	0.25	0.06–1.05	0.18	0.02–1.34
Substance abuse	**0.53**	**0.34–0.82**	0.72	0.38–1.34	0.56	0.31–1.00	0.62	0.27–1.43
Personality disorder	**0.61**	**0.50–0.74**	**0.68**	**0.53–0.86**	0.98	0.78–1.22	0.98	0.76–1.28
Dementia, organic brain syndrome, and intellectual disability	0.34	0.10–1.16	0.33	0.07–1.57	1.39	0.54–3.53	1.99	0.71–5.62

*Note:* Final model is adjusted with sociodemographic (gender, age, and family status) and socioeconomic (education, income, and occupation) factors, and with all the factors in the table.

Total *n* = 3,316 in the final multinomial logistic regression model.

Statistical significance *p* < 0.05 (bolded).

## Discussion

We studied medications taken in a nationally representative sample of CMD patients who had been granted a DP. The prevalence of taking the recommended medication peaked in the DP year and decreased thereafter. Alarmingly, 14.6% of our subjects were not taking antidepressants or antipsychotics during the study period, even though these, as monotherapy or in combination, are the treatment of choice in CMDs. This suboptimality of treatment of CMDs has also been reported earlier, both in Finland [51, 52] and globally [7]. Of socioeconomic factors, only income was associated with better adherence to antidepressant medication for over a year. We found no associations between SES and taking BZDR. Taking medication was not related to RTW. However, rehabilitation was positively associated, and comorbid personality disorders negatively associated, with RTW.

In contrast to existing studies [27–30, 53], we detected no SES differences in the propensity to take antidepressants. In our study, all subjects were suffering from CMD severe enough to warrant a DP, which may explain the more pronounced demand for antidepressants in all SES categories. Moreover, in the Finnish healthcare system, applying for a DP is contingent upon having taken antidepressants, because a DP may not be granted for inadequately treated health conditions. Considering this, it is remarkable that we found every seventh of the study subjects to be suffering from CMD and not taking antidepressants or antipsychotics. We found no SES factors or comorbidities to explain this. This is in line with earlier research [51, 52] indicating that the Finnish public healthcare system does not guarantee quality treatment for CMDs before applying for a DP. It is possible that a subgroup of patients with chronic CMD and other disease burden is deemed treatment-resistant through many inconclusive treatment trials. These people may navigate the system between short-term employments, sick leaves, and unemployment. After sickness benefit has been justified for a person for 300 working days and the capacity to rehabilitate is deemed low, a DP may be granted without any further requirement to try antidepressants. To minimize the negative effects related to DP for individuals and for society, it would be essential to ensure that all possible treatment modalities available are tried before granting the DP.

In contrast to earlier studies [37–40], a low level of education was not associated with non-adherence to medication, probably due to population-specific factors in our study. On the contrary, we found minor but important differences in antidepressant adherence due to income, and it is noteworthy that this association was seen despite the debilitating nature of CMDs. Being unable to afford medication has been found to be a risk factor for non-adherence [38]. Earlier research has shown that when the amount the patient must pay for medication is reduced, adherence, especially among the lowest income classes, improves [54]. This highlights the fact that economically disadvantaged people are more prone to not taking their medication. Despite the reimbursement system in Finland, personal responsibilities form a big share for low-income patients.

We found no SES differences in the sustained use of BZDR. Earlier research has reported some evidence that middle or low SES is linked to being prescribed BZDR and to long-term use [34–36]. Being on a DP has been shown to be a risk factor for persevering with BZDR [55]. Prolonged BZDR in our study was probably attributable to population-specific factors rather than to SES factors.

Prevalence of antipsychotic monotherapy continued to rise after DP, which may be explained by the fact that a comorbid schizophrenia spectrum or bipolar disorder determines the patient’s health condition more than CMD. These conditions may not be affected by alleviating the demands of working life.

We found no association between antidepressant adherence or long-term use of BZDR and RTW. Regarding antidepressants, this is in line with the literature [13, 21, 22]. Antidepressants have proven efficacious in treating CMD symptoms [4, 6, 15], but to regain functional capacity, rehabilitation seems essential [8, 12–14]. Regarding BZDR, an earlier study has reported a connection between being on BZDR long-term and labour market marginalization, possibly due to cognitive adverse effects [26, 56]. Our study population was characterized by long-term, chronic, or recurrently debilitating CMDs. Probably at this end of the spectrum, the severity of CMD and its effect on functional capacity was more important in determining the working ability than the duration of taking BZDR.

One noteworthy finding was that a comorbid personality disorder was associated with lower RTW. Moreover, research has shown that personality disorders and CMDs in combination are more likely to result in more severe occupational outcomes [57]. People with personality disorders have long-term dysfunctional patterns of inner experiences and behaviour, causing distress in many aspects of life. CMD is probably a part of the causal pathway for personality disorder patients towards work disability [57]. Early recognition and long-term rehabilitation would probably be needed to prevent DP in this patient group.

### Strengths and limitations

The study sample was nationally representative of this specific CMD patient group. Our register data had a good coverage for SES, sociodemographic, and disease-related data with long-term follow-up both before and after the DP. Taking medications was assessed with the previously validated PRE2DUP method [49]. We also concede several limitations. First, due to the register study setting, we cannot establish causal pathways between exposures and outcomes, but the implications of the results must be evaluated in the light of prior research. Furthermore, there are several underlying factors unreachable with our data, affecting, for example, an individual’s decisions to terminate antidepressant treatment, including tolerability, logistic factors, and system-related factors. Second, we made the research-based assumption of treatment adherence to antidepressants requiring more than a year of continuous use. Third, we had no data on neuromodulatory or ketamine treatments, which are also treatments for treatment-resistant depression. Fourth, given the wide variety of medications taken, we decided to use the longest individual period of taking medications to assess adherence. However, we also analysed regression models using the total days of medication taken during the study period, and the results were consistent, both regarding SES and RTW analyses, which supports our findings. Fifth, the data were from the period 2007–2015, which is over a decade ago. However, no major changes in the Finnish economic structure or unemployment rate have occurred since then, but the COVID-19 pandemic served to increase the demand for CMD treatments, which may have widened the treatment gap. However, this should not impair the generalizability of the results.

## Conclusion

Pharmacological treatment of debilitating CMDs before DP was found suboptimal at the group level in this register study, and this was related to factors other than socioeconomic factors. Of socioeconomic factors, only income was positively associated with antidepressant adherence. RTW was associated with factors other than medications taken, with rehabilitation positively and comorbid personality disorders negatively. The results underline the importance of optimizing CMD treatment regardless of SES, and the importance of rehabilitation along with medication contributing to return to work.

## Supporting information

10.1192/j.eurpsy.2025.10046.sm001 Leppänen et al. supplementary material Leppänen et al. supplementary material

## Data Availability

Register data were obtained from the registers of the Social Insurance Institution of Finland, the Finnish Centre for Pensions, the Finnish Institute for Health and Welfare, and Statistics Finland. The combined data were stored in the server of Statistics Finland. Restrictions apply to the availability of these data. The data were used under license for the current study and are not publicly available. More information on the data is available on request from the corresponding author.

## Supplementary material

The supplementary material for this article can be found at http://doi.org/10.1192/j.eurpsy.2025.10046.
