# Supplementary material for: Socioeconomic status, antidepressant use, and return to work after disability due to common mental disorders
Source: Eur Psychiatry. 2025 Jun 13;68(1):e79. doi: 10.1192/j.eurpsy.2025.10046 (PMC12188336; doi:10.1192/j.eurpsy.2025.10046)
Supplement: Leppänen et al. supplementary material [file S0924933825100461sup001.docx]

**Supplementary data**

Table I. Demographics of the study population (%) and longest duration of taking antidepressants during the six-year study period.

|  | None | 1–90 d | 91–365 d | Over a year | Total |
| --- | --- | --- | --- | --- | --- |
|  | 2436 (19.7) | 841 (6.8) | 2109 (17.0) | 7002 (56.5) | 12388 |
| Gender |  |  |  |  |  |
| Male | 888 (19.0) | 310 (6.6) | 835 (17.8) | 2645 (56.5) | 4678 |
| Female | 1548 (20.1) | 531 (6.9) | 1274 (16.5) | 4357 (56.5) | 7710 |
| Age |  |  |  |  |  |
| 18–25 years | 202 (20.6) | 61 (6.2) | 163 (16.6) | 554 (56.5) | 980 |
| 26–35 years | 284 (19.4) | 101 (6.9) | 240 (16.4) | 841 (57.4) | 1466 |
| 36–45 years | 447 (21.4) | 155 (7.4) | 320 (15.3) | 1167 (55.9) | 2089 |
| 46–55 years | 782 (19.7) | 281 (7.1) | 670 (16.9) | 2241 (56.4) | 3974 |
| 56–65 years | 721 (18.6) | 243 (6.3) | 716 (18.5) | 2199 (56.7) | 3879 |
| Family status |  |  |  |  |  |
| Living alone | 895 (19.7) | 310 (6.8) | 760 (16.7) | 2586 (56.8) | 4551 |
| Couple | 736 (19.2) | 266 (6.9) | 689 (17.9) | 2152 (56.0) | 3843 |
| Single parent | 254 (20.3) | 109 (8.7) | 190 (15.2) | 697 (55.8) | 1250 |
| Couple with children | 551 (20.1) | 156 (5.7) | 470 (17.1) | 1567 (57.1) | 2744 |
| Education |  |  |  |  |  |
| Higher degree tertiary | 148 (19.8) | 56 (7.5) | 129 (17.3) | 413 (55.4) | 746 |
| Lower degree tertiary | 143 (17.9) | 52 (6.5) | 129 (16.2) | 473 (59.3) | 797 |
| Short cycle tertiary | 330 (18.7) | 125 (7.1) | 328 (18.6) | 980 (55.6) | 1763 |
| Upper secondary level | 1130 (19.6) | 390 (6.8) | 959 (16.6) | 3294 (57.1) | 5773 |
| Basic level | 685 (20.7) | 218 (6.6) | 564 (17.0) | 1842 (55.7) | 3309 |
| Income^a^ |  |  |  |  |  |
| Highest | 271 (16.4) | 116 (7.0) | 288 (17.4) | 978 (59.2) | 1653 |
| Middle-higher | 356 (19.3) | 122 (6.6) | 306 (16.6) | 1059 (57.5) | 1843 |
| Middle | 445 (19.2) | 140 (6.0) | 432 (18.6) | 1300 (56.1) | 2317 |
| Middle-lower | 562 (20.2) | 185 (6.6) | 459 (16.5) | 1577 (56.7) | 2783 |
| Lowest | 749 (20.8) | 256 (7.1) | 595 (16.5) | 2003 (55.6) | 3603 |
| Occupation^b^ |  |  |  |  |  |
| Entrepreneur | 145 (18.8) | 43 (5.6) | 156 (20.3) | 426 (55.3) | 770 |
| Upper white-collar worker | 245 (18.1) | 105 (7.8) | 229 (17.0) | 771 (57.1) | 1350 |
| Lower white-collar worker | 637 (18.9) | 229 (6.8) | 575 (17.1) | 1931 (57.3) | 3372 |
| Student | 203 (20.3) | 69 (6.9) | 158 (15.8) | 568 (56.9) | 998 |
| Unemployed | 250 (21.0) | 85 (7.1) | 213 (17.9) | 645 (54.1) | 1193 |
| Blue-collar worker | 430 (19.3) | 139 (6.2) | 389 (17.4) | 1275 (57.1) | 2233 |
| Vocational rehabilitation |  |  |  |  |  |
| Yes | 1032 (19.4) | 371 (7.0) | 908 (17.1) | 3012 (56.6) | 5323 |
| No | 1404 (19.9) | 469 (6.6) | 1201 (17.0) | 3991 (56.5) | 7065 |
| Psychotherapy |  |  |  |  |  |
| Yes | 417 (20.1) | 129 (6.2) | 359 (17.3) | 1171 (56.4) | 2076 |
| No | 2019 (19.6) | 712 (6.9) | 1750 (17.0) | 5831 (56.5) | 10312 |
| Co-morbidity |  |  |  |  |  |
| Somatic Condition  (excluding musculoskeletal  disorders) | 255 (18.8) | 95 (7.0) | 224 (16.6) | 779 (57.6) | 1353 |
| Musculosceletal Disorder | 233 (18.1) | 90 (7.0) | 224 (17.4) | 738 (57.4) | 1285 |
| Psychotic disorder | 22 (17.6) | 10 (8.0) | 19 (15.2) | 74 (59.2) | 125 |
| Bipolar disorder | 7 (15.6) | 5 (11.1) | 6 (13.3) | 27 (60.0) | 45 |
| ADHD | 7 (14.3) | 4 (8.2) | 8 (16.3) | 30 (61.2) | 49 |
| Autism | 11 (23.9) | 4 (8.7) | 9 (19.6) | 22 (47.8) | 46 |
| Substance abuse | 67 (18.9) | 27 (7.6) | 58 (16.4) | 202 (57.1) | 354 |
| Personality disorder | 348 (20.2) | 111 (6.4) | 299 (17.4) | 963 (56.0) | 1721 |
| Dementia, organic brain  syndrome and intellectual  disability | 21 (23.3) | 6 (6.7) | 8 (8.9) | 55 (61.1) | 90 |
| Permanent DP |  |  |  |  |  |
| Yes | 1507 (19.1) | 520 (6.6) | 1365 (17.3) | 4504 (57.0) | 7896 |
| No | 929 (20.7) | 321 (7.1) | 744 (16.6) | 2498 (55.6) | 4492 |

^a^ Total n = 12,199
^b^ Total n = 9,916

Table II. Classification of medication available in the data.

| **Antidepressants** |  |
| --- | --- |
|  |  |
| SSRIs |  |
| N06AB03 | fluoxetine |
| N06AB04 | citalopram |
| N06AB05 | paroxetine |
| N06AB06 | sertraline |
| N06AB08 | fluvoxamine |
| N06AB10 | escitalopram |
| \|  \| \| --- \| \|  \| \|  \| \|  \| \|  \| \|  \| \|  \| \|  \| \|  \| \|  \| |  |
| SNRIs |  |
| N06AX16 | venlafaxine |
| N06AX17 | milnacipran |
| N06AX21 | duloxetine |
|  |  |
| Mirtazapine |  |
| N06AX11 | mirtazapine |
|  |  |
| Other antidepressants |  |
| N06AA04 | clomipramine |
| N06AA06 | trimipramine |
| N06AA09 | amitriptyline |
| N06AA10 | nortriptyline |
| N06AA12 | doxepin |
| N06AG02 | moclobemide |
| N06AX03 | mianserine |
| N06AX05 | trazodone |
| N06AX12 | bupropion |
| N06AX22 | agomelatine |
| N06AX26 | vortioxetine |
| N05AN01 | lithium |
|  |  |
| **BZDR** |  |
|  |  |
| Anxiolytics |  |
| N05BA01 | diazepam |
| N05BA02 | chlordiazepoxide |
| N05BA04 | oxazepam |
| N05BA06 | lorazepam |
| (N05BA09) | clobazam, antiepileptic – excluded |
| N03AE01 | clonazepam |
| N06CA01 | amitriptyline+chlordiazepoxide |
|  |  |
| Hypnotics |  |
| N05CD02 | nitrazepam |
| N05CD07 | temazepam |
| N05CD08 | midazolam |
| N05CF01 | zopiclone |
| N05CF02 | zolpidem |
| **Antipsychotics** |  |
| N05AA01 | chlorpromazine |
| N05AA02 | levomepromazine |
| N05AB01 | dixyrazine |
| N05AB03 | perphenazine |
| N05AC01 | periciazine |
| N05AC02 | thioridazine |
| N05AD01 | haloperidol |
| N05AD03 | melperone |
| N05AE03 | sertindole |
| N05AE04 | ziprasidone |
| N05AE05 | lurasidone |
| N05AF01 | flupentixol |
| N05AF03 | chlorprothixene |
| N05AF05 | zuclopenthixol |
| N05AG02 | pimozide |
| N05AH02 | clozapine |
| N05AH03 | olanzapine |
| N05AH04 | quetiapine |
| N05AH05 | asenapine |
| N05AL01 | sulpride |
| N05AX08 | risperidone |
| N05AX12 | aripiprazole |
| N05AX13 | paliperidone |

Table III. Most common antidepressant groups and mirtazapine, monotherapy and polytherapy, measured as average number of days taken by the study population during the six-year study period.

|  | Average antidepressant  use (days)/ 6 years | | % of antidepressant use | |
| --- | --- | --- | --- | --- |
| **Monotherapy** | **719** | **82.5** | |  |
| SSRI | 398 | 45.7 | |  |
| SNRI | 173 | 19.8 | |  |
| Mirtazapine | 65 | 7.5 | |  |
| Other | 83 | 9.5 | |  |
|  |  |  | |  |
| **Polytherapy** | **152** | **17.5** | |  |
| Combination of two antidepressants | 143 | 16.5 | |  |
| Combination of three or more antidepressants | 9 | 1.0 | |  |

Table IV. Most common antidepressants, monotherapy and polytherapy, measured as average number of days taken by the study population during the six-year study period.*

|  | Average antidepressant  use (days)/ 6 years | | % of antidepressant use |
| --- | --- | --- | --- |
| **Monotherapy** | |  |  |
| Citalopram | 130 | | 14.9 |
| Venlafaxin | 126 | | 14.4 |
| Escitalopram | 125 | | 14.3 |
| Mirtazapine | 65 | | 7.5 |
| Sertraline | 63 | | 7.2 |
| Fluoxetine | 44 | | 5.1 |
| Duloxetine | 44 | | 5.1 |
| Other | 122 | | 14.0 |
|  |  | |  |
| **Polytherapy** |  | |  |
| Venlafaxin, Mirtazapine | 20 | | 2.3 |
| Escitalopram, Mirtazapine | 15 | | 1.7 |
| Citalopram, Mirtazapine | 13 | | 1.5 |
| Other combinations | 104 | | 11.9 |

*The smallest reported is 5%, except the most common combinations. The rest are combined in other or other combinations.

Table V. Most common antipsychotics taken, measured as average number of days in use by the study population during the six-year study period.*

|  | Average antipsychotics  use (days)/ 6 years | | % of antipsychotics use | |  |
| --- | --- | --- | --- | --- | --- |
| Quetiapine | | | 215 | | 43.3 |
| Olanzapin | | | 54 | | 10.9 |
| Risperidone | | | 52 | | 10.5 |
| Other/combinations | | | 176 | | 35.4 |

*The smallest reported is 5%. The rest are combined in other/combinations.

Table VI. Most commonly taken BZDR, measured as average number of days taken by the study population during the 6-year study period, anxiolytics and hypnotics separately.

|  | Average BZDR use (days)/ 6 years | % of BZDR use |
| --- | --- | --- |
| Anxiolytics use  Oxazepam | 70 | 15.7 |
| Diazepam | 39 | 8.8 |
| Alprazolam | 33 | 7.5 |
| Clonazepam | 29 | 6.6 |
| Other | 32 | 7.1 |
|  |  |  |
| Hypnotics use |  |  |
| Zopiclone | 76 | 17.0 |
| Zolpidem | 35 | 7.8 |
| Other | 19 | 4.5 |
|  |  |  |
| BZDR combinations |  |  |
| All combinations | 104 | 25.0 |

*The smallest reported is 5% and the rest are combined in other/all combinations

Table VII. Socioeconomic and sociodemographic factors and rehabilitation associated with taking anxiolytics short-term (1-180 days) and long-term (over 180 days). The reference is no anxiolytics taken.

|  | 1–180 days | | | | Over 180 days | | | |
| --- | --- | --- | --- | --- | --- | --- | --- | --- |
|  | Crude model | | Final model | | Crude model | | Final model | |
|  | OR | 95 % CI | OR | 95 % CI | OR | 95 % CI | OR | 95 % CI |
| Gender |  |  |  |  |  |  |  |  |
| Male | 1.00 | 0.91–1.10 | 0.99 | 0.88–1.12 | 0.98 | 0.89–1.06 | 0.99 | 0.89–1.11 |
| Female (reference) |  |  | 1 |  |  |  | 1 |  |
| Age |  |  |  |  |  |  |  |  |
| 18–25 years | 0.91 | 0.76–1.10 | 0.89 | 0.68–1.17 | 0.89 | 0.75–1.06 | 0.89 | 0.70–1.14 |
| 26–35 years | 0.90 | 0.77–1.06 | 0.91 | 0.73–1.13 | 0.91 | 0.79–1.05 | 0.94 | 0.78–1.15 |
| 36–45 years | 0.91 | 0.79–1.05 | 1.00 | 0.83–1.21 | **0.88** | **0.77–1.00** | 0.88 | 0.74–1.04 |
| 46–55 years | 1.06 | 0.94–1.19 | 1.13 | 0.98–1.30 | 1.05 | 0.94–1.17 | 1.05 | 0.93–1.20 |
| 56–65 years (reference) | 1 |  | 1 |  | 1 |  | 1 |  |
| Family status |  |  |  |  |  |  |  |  |
| Living alone | 0.97 | 0.86–1.10 | 0.94 | 0.80–1.09 | 1.03 | 0.92–1.15 | 0.99 | 0.86–1.15 |
| Couple | 1.06 | 0.93–1.21 | 0.98 | 0.84–1.14 | **1.16** | **1.03–1.31** | 1.10 | 0.96–1.27 |
| Single parent | 0.98 | 0.82–1.17 | 0.91 | 0.73–1.13 | 1.17 | 0.91–1.26 | 1.07 | 0.88–1.29 |
| Couple with children (references) | 1 |  | 1 |  | 1 |  | 1 |  |
| Education |  |  |  |  |  |  |  |  |
| Higher degree tertiary | 0.96 | 0.78–1.19 | 0.88 | 0.67–1.16 | 1.04 | 0.86–1.26 | 0.99 | 0.77–1.28 |
| Lower degree tertiary | 1.02 | 0.83–1.25 | 0.94 | 0.73–1.21 | 1.08 | 0.90–1.30 | 1.11 | 0.89–1.40 |
| Short cycle tertiary | 0.94 | 0.81–1.10 | 0.84 | 0.69–1.02 | 1.00 | 0.86–1.15 | 0.92 | 0.77–1.09 |
| Upper secondary level | 0.94 | 0.84–1.05 | 0.89 | 0.77–1.02 | 1.04 | 0.94–1.16 | 0.96 | 0.85–1.10 |
| Basic level (reference) | 1 |  | 1 |  | 1 |  | 1 |  |
| Income |  |  |  |  |  |  |  |  |
| Highest | 1.15 | 0.99–1.34 | 1.22 | 0.97–1.52 | 1.14 | 0.99–1.31 | 1.01 | 0.82–1.25 |
| Middle-higher | 1.08 | 0.94–1.26 | 1.11 | 0.90–1.36 | 1.07 | 0.93–1.23 | 0.90 | 0.74–1.09 |
| Middle | 1.04 | 0.91–1.20 | 0.97 | 0.80–1.18 | 1.11 | 0.98–1.26 | 1.03 | 0.86–1.23 |
| Middle-lower | 0.97 | 0.85–1.11 | 0.94 | 0.78–1.12 | 1.12 | 1.00–1.27 | 1.03 | 0.88–1.22 |
| Lowest (reference) | 1 |  | 1 |  | 1 |  | 1 |  |
| Occupation |  |  |  |  |  |  |  |  |
| Entrepreneur | 1.10 | 0.89–1.37 | 1.06 | 0.84–1.34 | 1.08 | 0.89–1.31 | 1.06 | 0.86–1.31 |
| Upper white-collar worker | 1.07 | 0.90–1.28 | 1.01 | 0.81–1.26 | 1.02 | 0.86–1.20 | 0.99 | 0.80–1.21 |
| Lower white-collar worker | 1.03 | 0.89–1.19 | 1.03 | 0.88–1.21 | 1.05 | 0.92–1.19 | 1.05 | 0.91–1.21 |
| Student | 1.02 | 0.84–1.25 | 1.10 | 0.86–1.40 | 0.95 | 0.79–1.14 | 0.99 | 0.80–1.24 |
| Unemployed | 0.89 | 0.74–1.08 | 0.93 | 0.76–1.15 | 0.92 | 0.78–1.09 | 0.91 | 0.76–1.11 |
| Blue-collar worker (reference) | 1 |  | 1 |  | 1 |  | 1 |  |
| Co-morbidity |  |  |  |  |  |  |  |  |
| Somatic Condition (excl musculoskeletal disorders) | 1.08 | 0.93–1.26 | 1.07 | 0.89–1.28 | 1.13 | 0.99–1.29 | 1.12 | 0.95–1.31 |
| Musculosceletal Disorder | 1.03 | 0.89–1.20 | 1.03 | 0.85–1.24 | 1.01 | 0.88–1.17 | 0.97 | 0.82–1.16 |
| Psychotic disorder | 0.91 | 0.57–1.48 | 1.27 | 0.71–2.28 | 1.00 | 0.65–1.52 | 1.15 | 0.66–1.99 |
| Bipolar disorder | 1.36 | 0.64–2.88 | 0.88 | 0.29–2.70 | 1.37 | 0.69–2.73 | 1.55 | 0.67–3.59 |
| ADHD | 1.11 | 0.54–2.30 | 2.06 | 0.88–4.79 | 1.03 | 0.52–2.04 | 1.74 | 0.77–3.94 |
| Autism | 0.96 | 0.43–2.13 | 1.17 | 0.45–3.00 | 1.21 | 0.62–2.36 | 1.40 | 0.62–3.17 |
| Substance abuse | 0.90 | 0.68–1.20 | **0.54** | **0.34–0.88** | 0.98 | 0.76–1.27 | 0.98 | 0.60–1.39 |
| Personality disorder | 0.97 | 0.85–1.12 | 0.55 | 0.87–1.22 | 1.01 | 0.89–1.14 | 1.01 | 0.86–1.17 |
| Dementia, organic brain syndrome and intellectual  disability | 1.06 | 0.62–1.81 | 0.56 | 0.46–1.92 | 0.95 | 0.57–1.59 | 0.82 | 0.41–1.63 |
| Vocational rehabilitation |  |  |  |  |  |  |  |  |
| Yes | 1.02 | 0.93–1.13 | 1.02 | 0.91–1.15 | 0.97 | 0.89–1.06 | 0.98 | 0.88–1.09 |
| No (reference) | 1 |  | 1 |  | 1 |  | 1 |  |
| Psychotherapy |  |  |  |  |  |  |  |  |
| Yes | 1.13 | 0.99–1.28 | 1.10 | 0.94–1.28 | 1.04 | 0.93–1.16 | 0.97 | 0.84–1.11 |
| No (reference) | 1 |  | 1 |  | 1 |  | 1 |  |

Total n =9,075 in the adjusted multinomial logistic regression model

*Statistical significance *p* < 0.05

Table VIII. Socioeconomic and sociodemographic factors and rehabilitation associated with taking hypnotics short-term (1–180 days) and long-term (over 180 days). The reference is no hypnotics taken.

|  | 1–180 days | | | | Over 180 days | | | |
| --- | --- | --- | --- | --- | --- | --- | --- | --- |
|  | Crude model | | Final model | | Crude model | | Final model | |
|  | OR | 95 % CI | OR | 95 % CI | OR | 95 % CI | OR | 95 % CI |
| Gender |  |  |  |  |  |  |  |  |
| Male | 1.06 | 0.97–1.17 | 1.07 | 0.95–1.20 | 1.05 | 0.95–1.15 | 1.09 | 0.97–1.22 |
| Female (reference) |  |  | 1 |  |  |  | 1 |  |
| Age |  |  |  |  |  |  |  |  |
| 18–25 years | 0.99 | 0.83–1.19 | 1.14 | 0.88–1.48 | 0.83 | 0.69–1.01 | **0.76** | **0.58–0.99** |
| 26–35 years | 0.99 | 0.85–1.16 | 1.07 | 0.87–1.31 | 0.92 | 0.78–1.08 | **0.79** | **0.64–0.98** |
| 36–45 years | 0.96 | 0.84–1.10 | 1.06 | 0.89–1.27 | 0.87 | 0.75–1.00 | **0.81** | **0.68–0.98** |
| 46–55 years | 0.97 | 0.87–1.09 | 0.96 | 0.83–1.10 | 1.00 | 0.89–1.12 | 0.94 | 0.82–1.08 |
| 56–65 years (reference) | 1 |  | 1 |  | 1 |  | 1 |  |
| Family status |  |  |  |  |  |  |  |  |
| Living alone | 0.92 | 0.82–1.04 | 0.91 | 0.79–1.06 | 1.01 | 0.89–1.14 | 1.09 | 0.93–1.27 |
| Couple | 1.02 | 0.90–1.16 | 1.05 | 0.91–1.22 | 1.06 | 0.93–1.21 | 1.03 | 0.88–1.20 |
| Single parent | 0.90 | 0.76–1.08 | 0.87 | 0.70–1.07 | 1.04 | 0.87–1.24 | 1.01 | 0.82–1.25 |
| Couple with children (references) | 1 |  | 1 |  |  |  | 1 |  |
| Education |  |  |  |  |  |  |  |  |
| Higher degree tertiary | 1.01 | 0.82–1.23 | 1.09 | 0.83–1.43 | 0.95 | 0.77–1.17 | 0.90 | 0.68–1.19 |
| Lower degree tertiary | 1.03 | 0.84–1.26 | 1.01 | 0.79–1.30 | 1.10 | 0.90–1.34 | 1.14 | 0.90–1.45 |
| Short cycle tertiary | 0.87 | 0.75–1.01 | 0.91 | 0.75–1.10 | 0.91 | 0.78–1.06 | 0.86 | 0.71–1.04 |
| Upper secondary level | 1.01 | 0.91–1.13 | 0.99 | 0.86–1.13 | 0.90 | 0.81–1.01 | 0.87 | 0.76–1.00 |
| Basic level (reference) | 1 |  | 1 |  | 1 |  | 1 |  |
| Income |  |  |  |  |  |  |  |  |
| Highest | 1.07 | 0.92–1.24 | 0.95 | 0.77–1.19 | 1.10 | 0.95–1.29 | 0.98 | 0.78–1.23 |
| Middle-higher | 0.97 | 0.84–1.13 | 0.88 | 0.71–1.08 | **1.20** | **1.03–1.38** | 1.06 | 0.86–1.30 |
| Middle | 1.06 | 0.93–1.21 | 1.00 | 0.83–1.21 | 1.07 | 0.93–1.23 | 0.98 | 0.81–1.19 |
| Middle-lower | 1.09 | 0.96–1.24 | 0.96 | 0.81–1.15 | 1.06 | 0.93–1.21 | 1.04 | 0.87–1.25 |
| Lowest (reference) | 1 |  | 1 |  | 1 |  | 1 |  |
| Occupation |  |  |  |  |  |  |  |  |
| Entrepreneur | 0.97 | 0.78–1.20 | 0.98 | 0.78–1.23 | 1.07 | 0.87–1.33 | 1.06 | 0.84–1.33 |
| Upper white-collar worker | 0.91 | 0.76–1.08 | 0.93 | 0.75–1.16 | 0.97 | 0.81–1.16 | 0.94 | 0.75–1.17 |
| Lower white-collar worker | 0.91 | 0.80–1.05 | 1.00 | 0.86–1.17 | 1.03 | 0.90–1.19 | 1.07 | 0.91–1.25 |
| Student | 0.84 | 0.69–1.02 | 0.83 | 0.65–1.05 | 0.98 | 0.81–1.19 | 1.06 | 0.84–1.35 |
| Unemployed | 0.92 | 0.77–1.10 | 0.93 | 0.76–1.13 | 0.90 | 0.74–1.08 | 0.89 | 0.72–1.10 |
| Blue-collar worker (reference) | 1 |  | 1 |  | 1 |  | 1 |  |
| Co-morbidity |  |  |  |  |  |  |  |  |
| Somatic Condition (excl musculoskeletal  disorders) | **1.29** | **1.12–1.48** | **1.32** | **1.12–1.57** | 1.10 | 0.94–1.27 | 1.02 | 0.85–1.21 |
| Musculosceletal Disorder | 1.03 | 0.89–1.19 | 1.05 | 0.88–1.26 | **0.85** | **0.72–0.99** | **0.77** | **0.64–0.94** |
| Psychotic disorder | 1.07 | 0.69–1.66 | 1.34 | 0.77–2.34 | 0.83 | 0.51–1.36 | 0.94 | 0.50–1.75 |
| Bipolar disorder | 1.55 | 0.79–3.01 | 2.00 | 0.87–4.57 | 0.75 | 0.31–1.82 | 0.64 | 0.18–2.21 |
| ADHD | 1.32 | 0.67–2.61 | **2.68** | **1.23–5.84** | 1.04 | 0.49–2.22 | 1.66 | 0.66–4.14 |
| Autism | 1.03 | 0.48–2.19 | 1.40 | 0.57–3.45 | 1.20 | 0.58–2.49 | 1.99 | 0.87–4.56 |
| Substance abuse | 1.07 | 0.82–1.40 | 0.95 | 0.64–1.40 | 0.87 | 0.65–1.17 | 0.74 | 0.48–1.12 |
| Personality disorder | 0.90 | 0.79–1.03 | 1.00 | 0.85–1.18 | 0.94 | 0.82–1.08 | 0.95 | 0.80–1.12 |
| Dementia, organic brain syndrome and  intellectual disability | 1.05 | 0.61–1.79 | 0.80 | 0.38–1.67 | 1.17 | 0.69–1.97 | 0.80 | 0.38–1.67 |
| Vocational rehabilitation |  |  |  |  |  |  |  |  |
| Yes | 0.99 | 0.90–1.10 | 0.96 | 0.86–1.07 | 1.03 | 0.94–1.14 | 1.02 | 0.91–1.14 |
| No (reference) | 1 |  | 1 |  | 1 |  | 1 |  |
| Psychotherapy |  |  |  |  |  |  |  |  |
| Yes | 1.10 | 0.97–1.25 | 1.07 | 0.92–1.25 | 1.02 | 0.90–1.15 | 0.97 | 0.83–1.13 |
| No (reference) | 1 |  | 1 |  | 1 |  | 1 |  |

Total n =9,075 in the adjusted multinomial logistic regression model

*Statistical significance *p* < 0.05


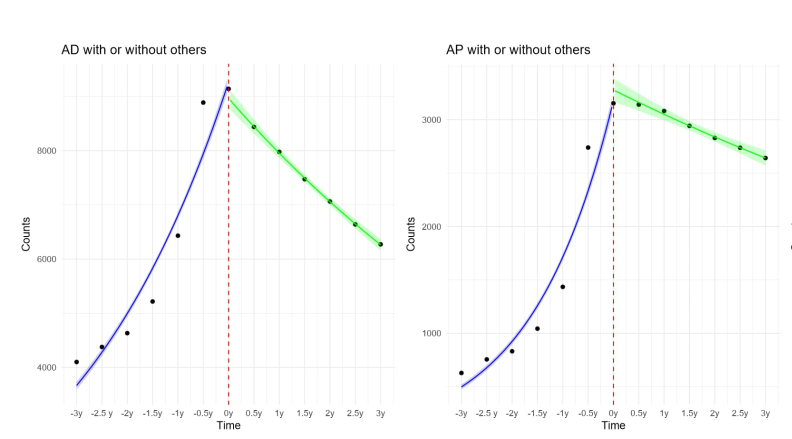


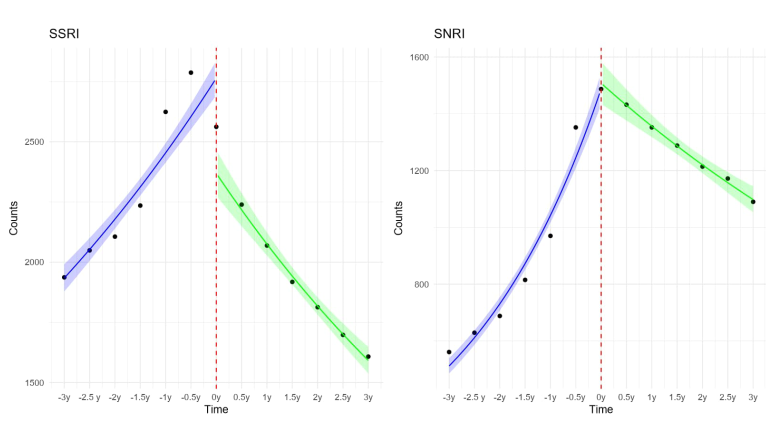


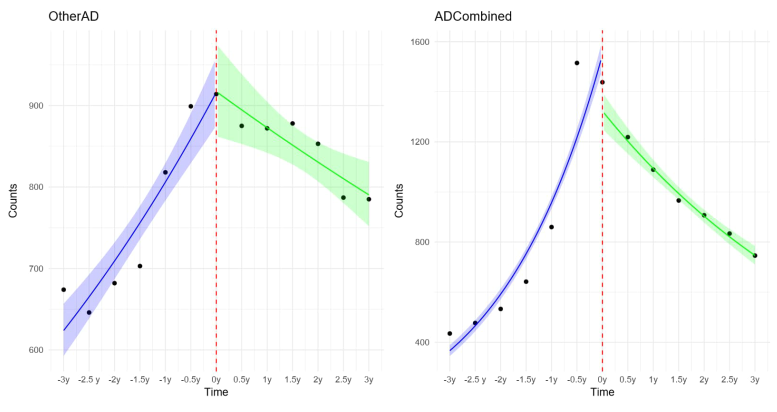


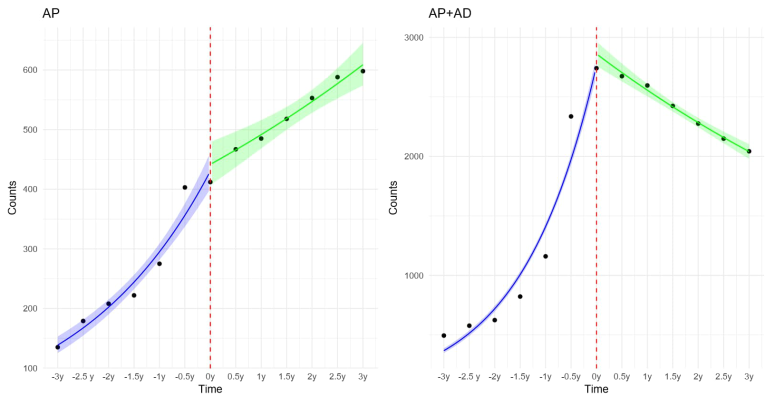


Figure I. Poisson interrupted time series analysis of medication taken three years before and after DP.

AD=antidepressants

AP=antipsychotics
